# Dielectric Response of Crosslinked *Adenanthera pavonina* L. Galactomannan in pH-Controlled Medium

**DOI:** 10.3390/polym17070954

**Published:** 2025-03-31

**Authors:** Antônia Millena de Oliveira Lima, Fernando Mendes, Lincoln Almeida Cavalcante, Cristiane Carvalho Araújo, Beatriz da Silva Batista, João Pedro Lemos Morais, Filipe Miguel Borges Amaral, Ana Angélica Mathias Macêdo

**Affiliations:** 1 Laboratório de Pesquisa, Instituto Federal do Maranhão, Campus Imperatriz, Imperatriz 65906-335, Brazil; millena.lima@bolsista.ifma.edu.br (A.M.d.O.L.); pg55653@uem.br (L.A.C.); carvalhoaraujo@acad.ifma.edu.br (C.C.A.); jplmorais@ufmg.br (J.P.L.M.); ana.angelica@ifma.edu.br (A.A.M.M.); 2 Polytechnic University of Coimbra, Rua da Misericórdia, Lagar dos Cortiços, S. Martinho do Bispo, 3045-093 Coimbra, Portugal; fjmendes@estesc.ipc.pt; 3 H&TRC—Health & Technology Research Center, Coimbra Health School, Polytechnic University of Coimbra, Rua 5 de Outubro, 3045-043, Coimbra, Portugal; 4 Biophysics Institute of Faculty of Medicine, Coimbra Institute for Clinical and Biomedical Research (iCBR) Area of Environment Genetics and Oncobiology (CIMAGO), University of Coimbra, 3000-548 Coimbra, Portugal; 5 Center for Innovative Biomedicine and Biotechnology, University of Coimbra, 3004-504 Coimbra, Portugal; 6 European Association of Biomedical Scientists, B-1000 Brussels, Belgium; 7 Unidade Avançada, Centro de Ciências Sociais, Saúde e Tecnológica, Universidade Federal do Maranhão, Imperatriz 65900-410, Brazil; beatriz.batista@discente.ufma.br; 8 I3N and Physics Department, Aveiro University, 3810-193 Aveiro, Portugal

**Keywords:** galactomannan, crosslinking, glutaraldehyde, pH variation, physicochemical properties

## Abstract

This research investigates the production of galactomannan from *Adenanthera pavonina* L. in its crude form and its subsequent crosslinking with glutaraldehyde under various pH conditions. The study involved the creation of films and sponges from these materials, followed by a comprehensive analysis of their structural, thermal, swelling, and electrical properties. Galactomannan was crosslinked with a fixed concentration of 0.2 mol/L of glutaraldehyde, with pH levels ranging from 3 to 7. These films and sponges were prepared through a slow solvent evaporation process. The research encompassed multiple analytical techniques, including Fourier transform infrared spectroscopy, X-ray diffraction, thermogravimetry, swelling profile assessments, and impedance spectroscopy. The findings from structural analysis indicated that variations in pH did not alter the amorphous nature of the samples but did influence the interactions between galactomannan molecules and restricted the mobility of polymeric chains, which resulted in different dielectric responses. Crosslinked samples exhibited reduced water solubility compared to unprocessed galactomannan. Crosslinking also decreases the ability of the material to polarize and align in response to the electric field, which justifies why crosslinked samples present a lower dielectric constant than the crude sample.

## 1. Introduction

Galactomannans (Gals) are storage polysaccharides located in the cell wall of various plant seeds, with their main function being to act as an energy reserve and for water retention [1,2]. They are usually found in legume endosperms and consist of a linear chain of mannose units joined by β-(1→4) linkages, branched by D-galactose units joined by α-(1→6) linkages [3]. Due to its non-toxicity, Gal is applied in food engineering [4,5], tissue engineering [6], nanomaterials [7], and pharmaceutic products [8,9].

*Adenanthera pavonina* L. seeds, in particular, are rich, renewable, and unconventional sources of Gal, presenting antibacterial [10], anthelmintic [11], and anti-inflammatory [12] properties. Recent studies show that Gal obtained from *Adenanthera pavonina* L. exhibits coherent physicochemical properties for food and pharmaceutical use, such as non-toxicity, antioxidant activity, photostability under ultraviolet irradiation [13], antidiabetic activity [14], and antiviral properties [15].

Gal has also been shown to promote wound healing, making it a potential candidate for use in wound dressings [16]. However, its high solubility in water presents a significant limitation, restricting its effectiveness in such applications. To address this issue, Gal can be crosslinked into a three-dimensional polymeric network, enhancing its mechanical stability through the formation of crosslinks along the polymeric chain [17,18,19].

Among the various crosslinking methods, chemical crosslinking utilizes chemical agents such as borate [20], N,N-methylenebisacrylamide [21], citric acid [22], and others to facilitate the crosslinking of galactomannan with other materials. However, some of these agents exhibit toxicity, rendering the resulting hydrogel unsuitable for biomedical and food applications [23]. In contrast, glutaraldehyde (GA) is a low-cost crosslinking agent with relatively low toxicity and a high binding capacity, enabling the formation of stronger and more stable crosslinking bonds [5,24,25].

Despite its strong crosslinking capability, glutaraldehyde does not exist solely in its monomeric form in aqueous solutions. As reported by numerous authors in the literature, GA can present multiple structural forms, which can be modified by variations in the pH of the medium and may affect the crosslinking process. However, in diluted solutions with pH values between 3 and 8, the monomeric form of GA tends to predominate over its polymerized counterparts, highlighting the importance of pH control during crosslinking [26].

The pH of the medium plays a crucial role in the crosslinking process, as demonstrated by Hospodiuk et al. [27] and observed in our previous studies [28]. Variations in pH during the crosslinking of galactomannan have been shown to influence its swelling profile, suggesting that pH control can be utilized to adjust the physicochemical properties of these hydrogels. However, comprehensive studies on the structural, thermal, and electrical property changes of glutaraldehyde-crosslinked galactomannan as a function of pH have not been previously reported.

Galactomannan can be utilized in biomedical applications following appropriate polarization treatment, in addition to its established role in food production. Consequently, assessing the dielectric behavior of this raw material is essential, particularly by analyzing its response to radio frequencies and microwave heating. Devesa et al. [29] investigated the dielectric properties of both crude and ethanol-purified galactomannan, demonstrating that the purified galactomannan solution exhibits stable conductivity. This characteristic renders it highly suitable for applications in biosensing, biomedical fields, and the food industry [30].

Despite the promising applications of Gal solutions, it is essential to assess the electrical properties of galactomannan in different formats, such as crosslinked structures. To date, the authors have not identified any studies that examine the electrical properties of galactomannan crosslinked with glutaraldehyde.

From this perspective, the present study aims to analyze the crosslinking of galactomannan with glutaraldehyde and investigate the influence of different pH values on the crosslinking reaction. This analysis is conducted through the evaluation of structural properties, thermogravimetric behavior, swelling profiles, and electrical characteristics of both crude and crosslinked galactomannan. To facilitate these characterizations, films and sponges were prepared using crude galactomannan (Gal) as well as its crosslinked form with glutaraldehyde.

## 2. Materials and Methods

### 2.1. Materials

Seeds of *Adenanthera pavonina* L. were collected in Imperatriz, Maranhão, Brazil. Glutaraldehyde (C_5_H_8_O_2_) was purchased from *Êxodo Científica*.

### 2.2. Obtaining Galactomannan from Adenanthera pavonina L. Seeds

Gal was separated from *Adenanthera pavonina* L. seeds. Initially, the seeds were collected, selected, cleaned, heated to a temperature of 100 °C for 30 min, and taken to intumescence for 24 h in distilled water. Then, the seeds’ endosperms were manually separated, washed in distilled water, freeze-dried, and pulverized.

The primary physicochemical properties used to characterize crude galactomannan (Gal) obtained from *Adenanthera pavonina* L. include the mannose-to-galactose (M:G) ratio, polysaccharide composition, molecular mass, and viscosity. Reported M:G values for crude *Adenanthera pavonina* L. Gal range from 1.35:1 to 1.8:1 [31], with variations between 1.5:1 and 1.7:1 observed in sequential separations from the seed endosperm [32]. Factors such as the maturation stage of *Adenanthera pavonina* L. seeds, cultivation conditions, and differences in separation and purification methodologies can influence the M:G ratio, contributing to the variability reported in the literature [31].

The reported polysaccharide composition and viscosity properties (average intrinsic viscosity, Huggins coefficient (*k_H_*), viscosity average molecular mass (***M_v_***)) of *Adenanthera pavonina* L. crude Gal are given in Table 1 [31].

### 2.3. Galactomannan Crosslinking

Gal was crosslinked using GA as a crosslinking agent. Initially, a GA solution was prepared with a concentration of 0.2 mol/L, separated in three 200 mL aliquots, and its pH was adjusted to 3, 5, and 7 using HCl and NaOH solutions. Then, Gal powder was added to each aliquot to form a solution with a concentration of 2% Gal.

The solutions were stirred for 24 h for homogenization and centrifuged at 1308 G for 30 min at room temperature. The crosslinked Gal (GP) samples made at pH 3, 5, and 7 were named GP3, GP5, and GP7, respectively. Control samples were prepared by dissolving crude Gal (GB)—without crosslinking—in distilled water at 2% Gal concentration.

### 2.4. Preparation of Crosslinked Galactomannan Films and Sponges

Films and sponges were synthesized using crosslinked galactomannan. The films were obtained by slow solvent evaporation in a temperature-controlled environment. During the sponge preparation, the material was molded in Petri dishes, frozen for 24 h, and freeze-dried for 48 h for moisture removal.

### 2.5. Characterization

Fourier transform infrared (FTIR) spectra were obtained with a Fourier transform spectrometer (Vertex 70V, Bruker, Billerica, USA). The infrared transmission technique was used in the analyses, using potassium bromide (KBr) pellets as a matrix for the crude and crosslinked Gal films. The characterization was performed in the spectral region from 4000 to 400 cm^−1^, with 64 scans and a spectral resolution of 4 cm^−1^. The background was carried out using a KBr-only pellet.

X-ray diffraction (XRD) patterns were obtained for the crude and crosslinked samples in powder form using an X-ray diffractometer (Miniflex II, Rigaku, Tokyo, Japan) with a 30 kV voltage and 15 mA current.

Thermogravimetry (TG) and derivative thermogravimetry (DTG) measurements were performed with a thermogravimetric analyzer (DTG-60, Shimadzu, Kyoto, Japan). Crude and crosslinked samples in powder form were placed in a closed aluminum crucible in a nitrogen atmosphere with a flow rate of 100 mL/min. The temperature range of TG and DTG measurements was from 25 to 595 °C, with a heating rate of 10 °C/min.

For the dielectric analysis, the GB sample and crosslinked Gal samples (GP3, GP5, and GP7) were powdered and pelletized in a bulk cylindrical shape. Impedance data of samples were obtained by the Solartron SI 1260 Impedance equipment, in the following conditions: frequency range of 100 Hz–1 MHz, 1 V signal amplitude, and at room temperature. Copper electrodes were used to directly contact with the samples’ surface.

The crude Gal and crosslinked GP sponges (~0.001 g) were immersed in 50 mL of distilled water for up to 90 min at room temperature (~25 °C). At every 10 min, the excess water was removed, and the sponges’ mass was adequately weighed on an analytical balance at room temperature. The swelling degree (Q) was calculated using Equation (1), adapted from Bueno et al. [33], where M is the mass of the swollen sample and *m* is the initial mass.

(1) $$Q\;=\frac{M-m}{m}$$

## 3. Results and Discussion

### 3.1. FTIR Analysis

**Figure 1:**
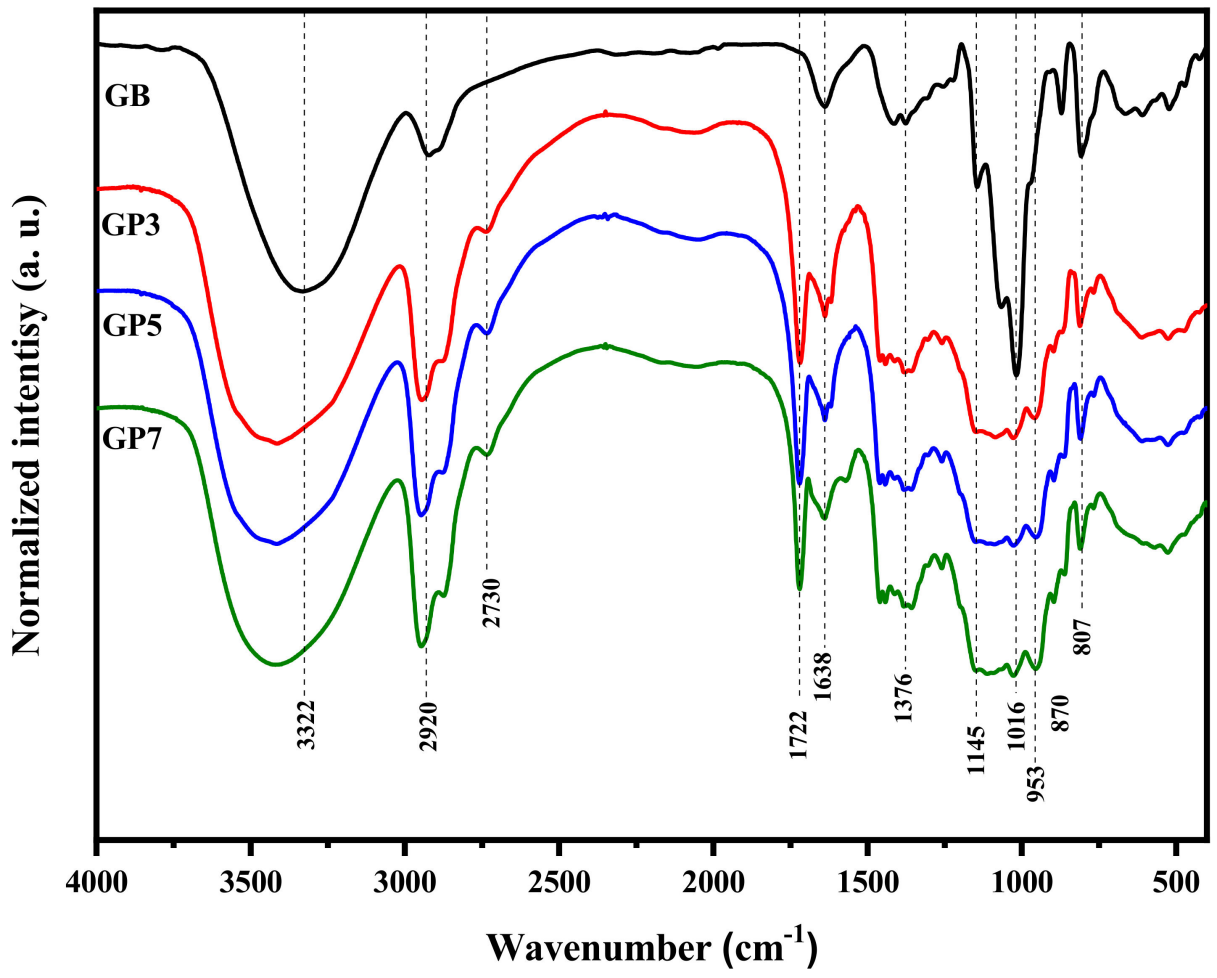
FTIR spectra of GB, GP3, GP5, and GP7 samples.

Figure 1 shows the FTIR spectra of GB and crosslinked samples (GP3, GP5, and GP7), with the wavenumber and associated functional group of each absorption band. The bands centered at 807 and 870 cm^−1^ refer to the α-D-galactose and β-D-mannose bonds units, respectively, of the galactomannan structure [34,35]. In addition, the band between 950 and 970 cm^−1^ (centered at 953 cm^−1^) is attributed to the axial deformation of the C-OH bond of Carbon 4 [34].

The bands localized in the region between 920 and 1200 cm^−1^ are associated with the stretching vibration of the C-OH, C-O-C, and -C-C-O groups of the polymer structure. Specifically, the band at 1160 cm^−1^ can be attributed to the bending of the δ(C-O) vibrational angular stress modulus due to the pyranose ring. In addition, the bands referring to stretching in the galactose and mannose rings appear between 1620 and 1657 cm^−1^ [36,37,38].

The band around 1722 cm^−1^ appeared after the crosslinking process. This band can be referred to as the vibrational mode of the carbonyl (C=O) of the aldehyde peak (CHO) of the unreacted glutaraldehyde molecules [39]. The increase in the pH of the media does not change the intensity of this band.

The region between 2930 and 2940 cm^−1^ is attributed to the stretching vibration of the C-H bond of the CH_2_ group, which is supported by the bands between 1350 and 1450 cm^−1^, attributed to symmetric deformations of the CH_2_ and CH groups [40]. The band centered at 2730 cm^−1^, shown for crosslinked samples, is a duplet absorption with a 2920 cm^−1^ peak and can be attributed to the C–H stretching of the aldehydes [41]. A slight shift was observed for the bands at 2920 cm^−1^ and 3420 cm^−1^ after the crosslinking. The band around 3420 cm^−1^ corresponds to the O–H stretching vibrations of hydroxyl groups in galactomannan. After crosslinking with glutaraldehyde, some hydroxyl groups react to form acetal or hemiacetal linkages, reducing the availability of free –OH groups [20,37,42]. The band at ~2920 cm⁻^1^ is attributed to C–H stretching vibrations in the polysaccharide backbone. In this case, the crosslinking introduces new covalent bonds (acetal bridges), which modify the electronic environment around C–H groups, which slightly changes the vibrational response.

**Scheme 1:**
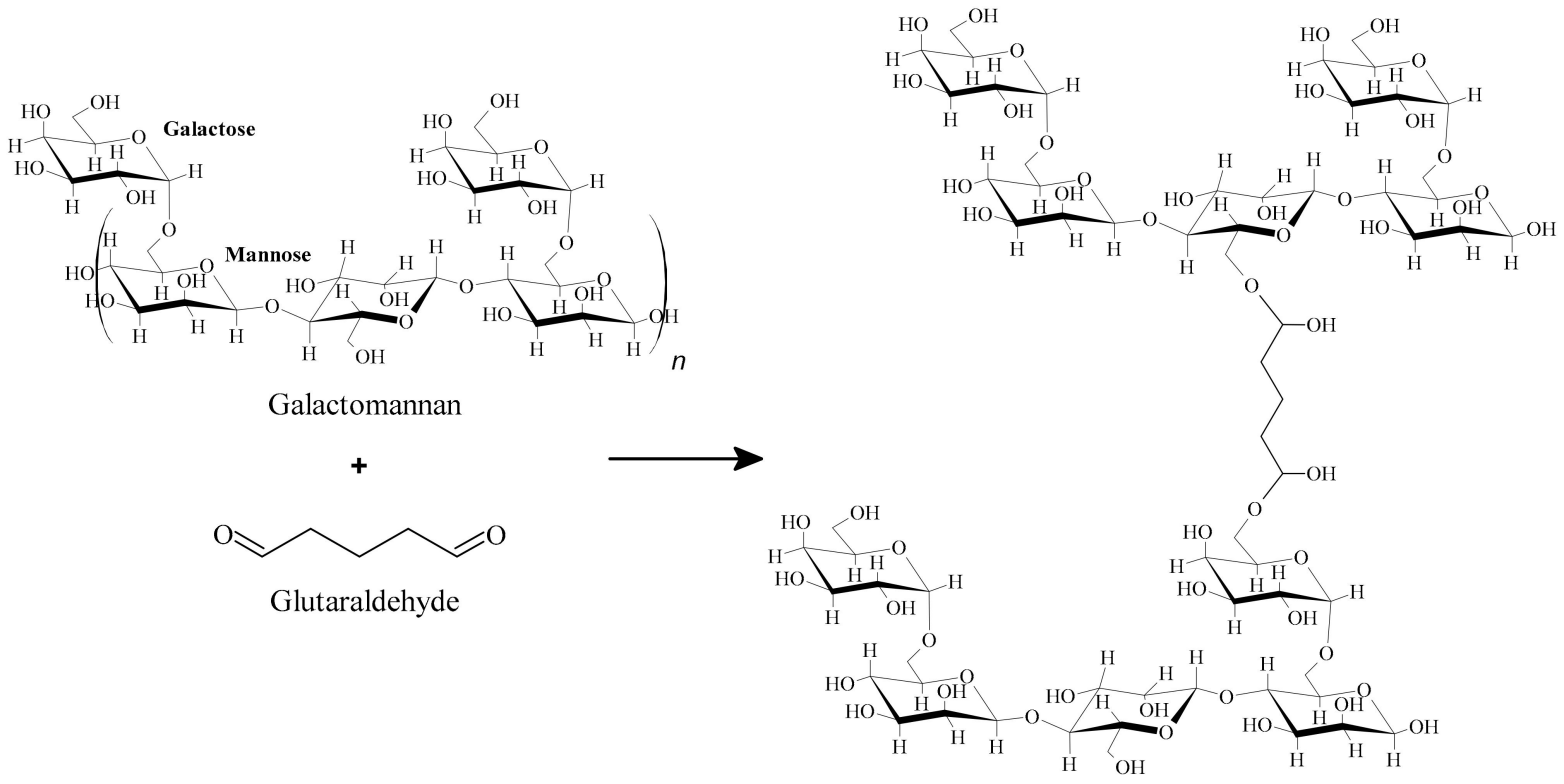
Crosslinking process of galactomannan using glutaraldehyde as crosslinker.

From FTIR analysis, we can state that regardless of the crosslinking with GA and the pH of the medium, all samples presented the characteristic functional groups of galactomannan: α-D-galactose and β-D-mannose. The data suggest that pH does not change the chemical structure of the samples because the characteristic functional groups of galactomannan remain the same. The crosslinking reaction of Gal with GA is presented in Scheme 1.

### 3.2. X-Ray Diffraction

**Figure 2:**
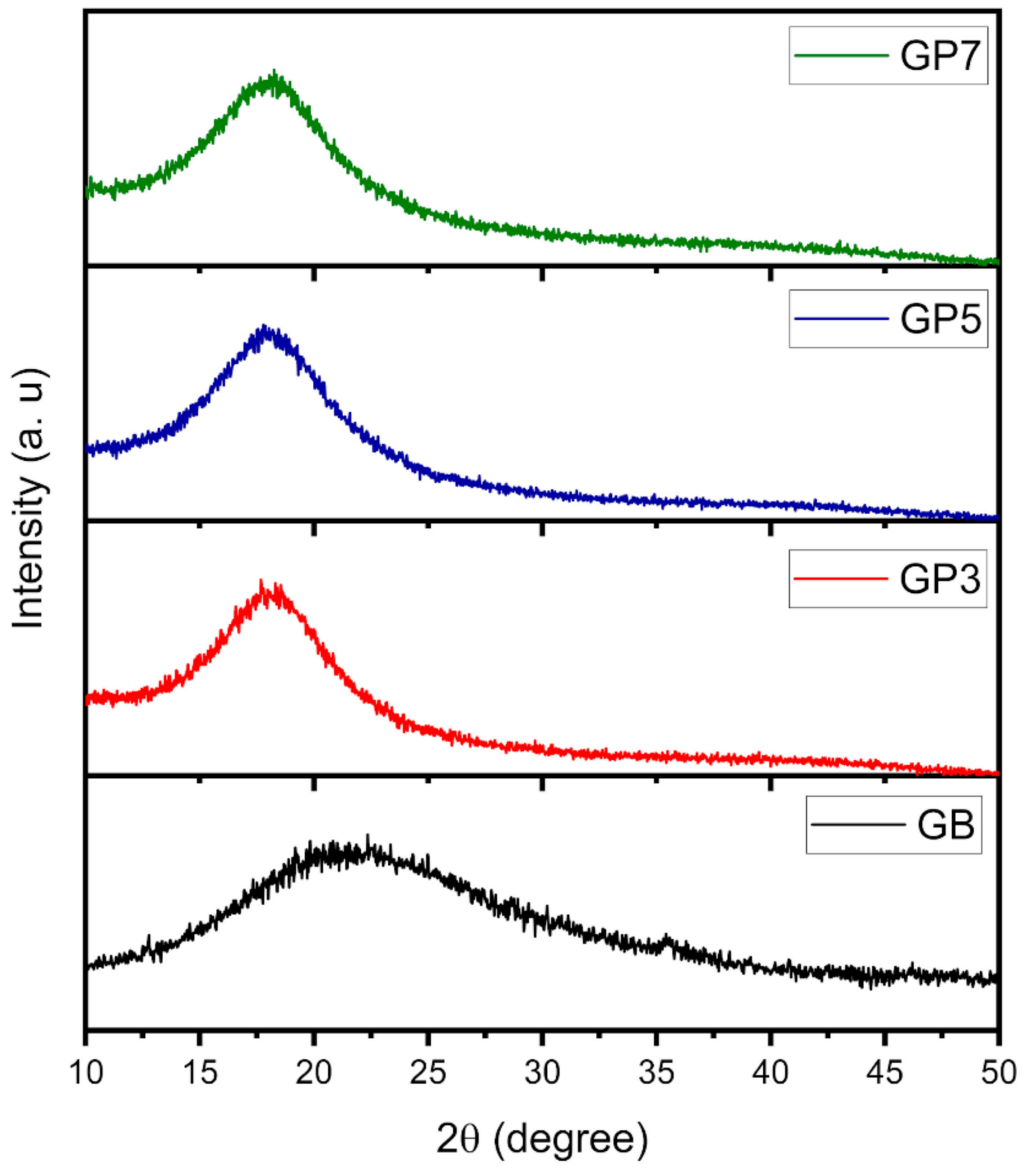
X-ray diffraction pattern of GB, GP3, GP5, and GP7 samples.

The amorphous nature of the samples was confirmed by the presence of a broad band in the diffractogram (Figure 2). The amorphous conformation was also observed for other polysaccharides, such as cashew gums [43], guar [44], Arabic [45], and karaya [46]. It is observed that the pH modification did not cause changes in the amorphous character of the crosslinked samples. However, it is observed that a shift and a narrowing of the amorphous halos occurred after the crosslinked process.

The full width at half maximum (FWHM) for all diffractograms was determined. A decrease in FWHM was observed from 14.8 to 5.9 for GB and GP7, respectively. This can be explained by the lower flexibility and higher packing of the polymer chains in the samples that underwent the crosslinking process. This behavior corresponds to the decrease in the intensity of the bands in the FTIR spectrum after the crosslinking process.

### 3.3. Thermogravimetric Analysis

**Figure 3:**
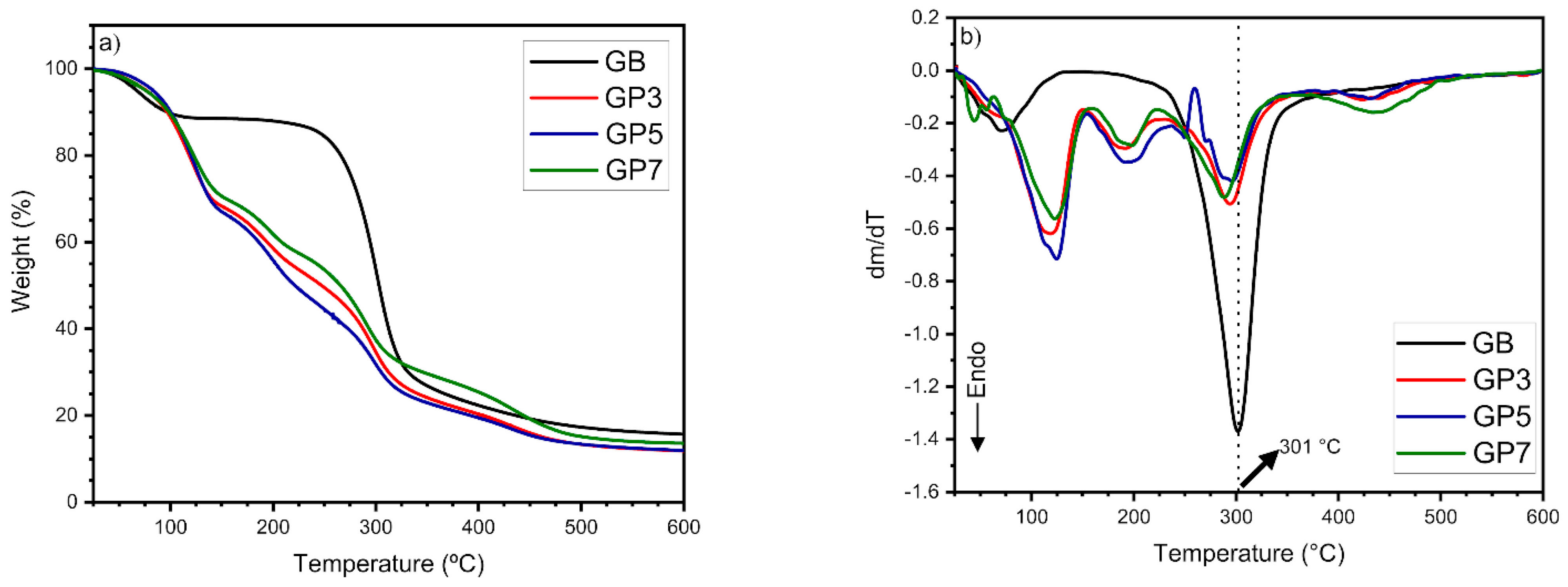
(**a**) TG and (**b**) DTG curves of GB, GP3, GP5, and GP7 samples.

Thermogravimetric analysis can identify thermal phenomena associated with mass variation as a function of temperature. Figure 3 shows TG/DTG curves for GB, GP3, GP5, and GP7 samples and Table 2 exhibits the percentage of weight loss and waste. The DTG curves revealed four weight loss events, as reported by Jamir and collaborators [47] and by Singh and Bothara [48]. The data suggest a multistep decomposition process, more evident for the crosslinked samples.

The first mass loss (ΔW1) for all samples between 50 and 150 °C is due to water evaporation and is directly associated with the nature of the functional groups, according to the literature [49,50]. GP3, GP5, and GP7 presented a similar weight loss of 32.25% to 34.14%. On the other hand, the GB sample showed a 11.45% weight loss. It occurs due to the crosslinking process and the shape of the samples because once they are freeze-dried, the samples acquire a spongy appearance with greater potential for liquid adsorption.

The second thermal event for GB between 250 and 450 °C, centered at 301 °C, is associated with the decomposition of the galactomannan structure and it corresponds to 71.95% weight (ΔW2). However, the second event for the crosslinked samples, centered at 193 °C, is associated with retained water [3,50]. In addition, this peak can occur due to the shrinkage of hydrogel after the dehydration process [51].

The third thermal event for GP3, GP5, and GP7 samples occurs in the range from 214 °C to 380 °C and is associated with the pyrolysis of the polysaccharide main chain, which results in a 29.11%, 20.85%, and 23.11% weight loss (ΔW3), respectively [50,52]. This large difference between crosslinked samples and GB can be associated with the increase in the organization of the polymer structure after the crosslinking process, seen in XRD patterns.

The peak at 301 °C, corresponding to polymer degradation, presents a shift as a function of the pH medium. This behavior can be explained by the increase in hydrogen bonds and free volume. The breaking of these bonds increases the molecular mobility, decreasing the glass transition temperature (Tg) of the materials [53]. This observation suggests a slight decrease in the thermal stability of Gal after glutaraldehyde crosslinking.

For crosslinked samples, a fourth event was identified, starting near 410 °C, which can be attributed to the further degradation of the polymer together with that of other components present in the sample, as reported in the literature [50,52].

The lowest residue content and highest weight loss were observed for GB, given the fact that, in this case, there were no modifications caused by reticulation in its structure. In addition, the degradation sequence that occurs in these polysaccharides starts with the elimination of adsorbed water followed by the release of coordination water and then linear chain fragments.

These values agree with those reported by Cerqueira and collaborators [3] for *Adenanthera pavonina* L., with values between 309.8 and 320.6 for polymer degradation, with an average residue content of 19.9%. It was observed that the thermal decomposition patterns of the samples show good thermal stability. Although GB presents greater thermal stability than the modified samples, both present acceptable responses.

### 3.4. Swelling Degree

**Figure 4:**
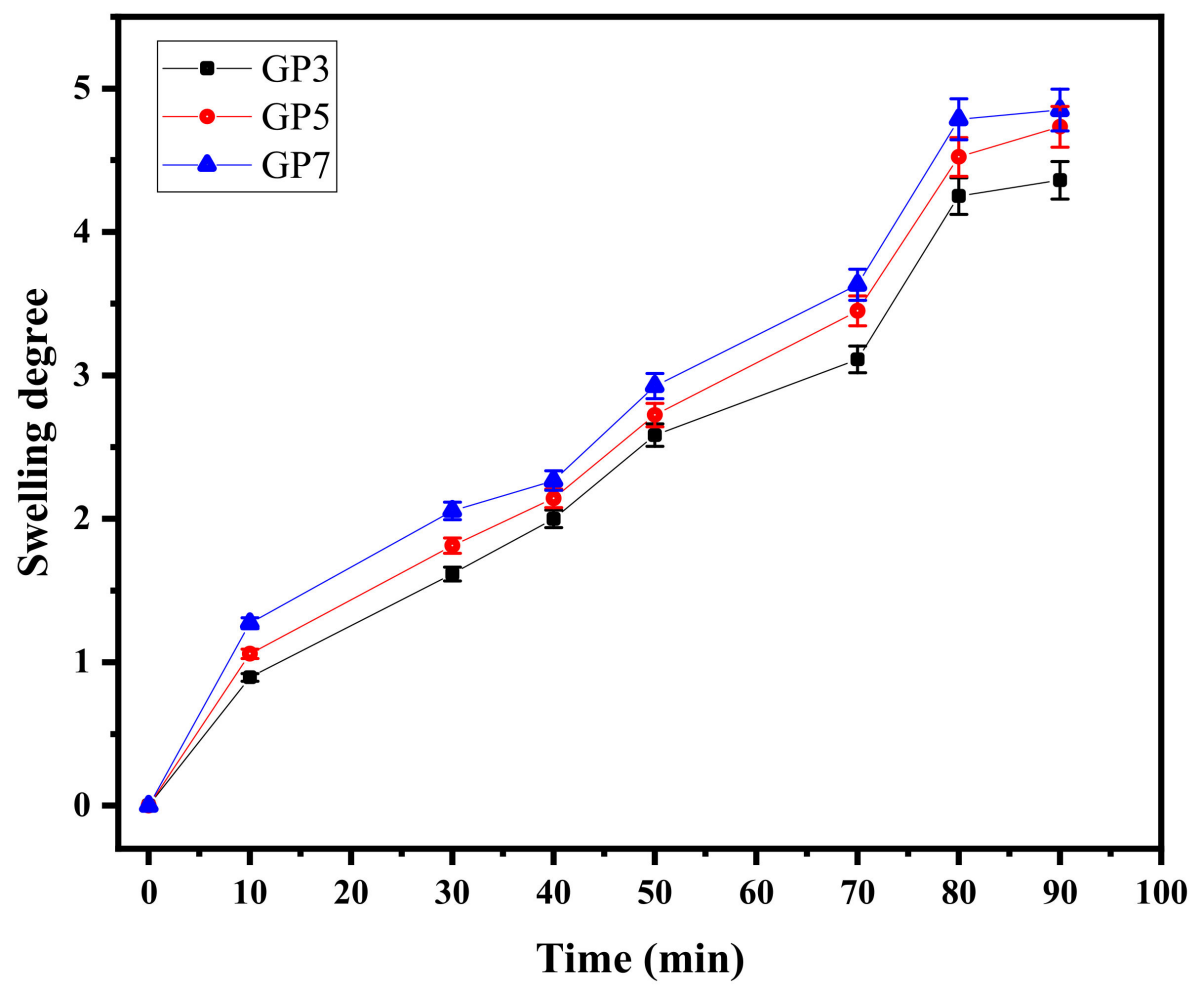
Swelling degree as a function of time for GP3, GP5, and GP7 samples. The lines are a guide to the eyes.

Figure 4 shows the swelling profile in water of GP3, GP5, and GP7 samples up to 90 min at room temperature, using a time interval of 10 min. The GB sample showed no swelling capacity and solubilized. Meanwhile, the solubility rate decreased with the crosslinking process for all the crosslinked samples, as it inhibits the mobility of the polymer chains [54].

An increase is observed in swelling capacity as a function of increasing pH. At a lower pH of the medium, glutaraldehyde tends to exist in a hydrated or polymerized form (cyclic hemiacetals and oligomers). While hydration reduces the availability of free aldehyde groups, acidic conditions can still promote the formation of hemiacetal or acetal linkages between glutaraldehyde and the hydroxyl groups of galactomannan. This process enhances crosslinking, leading to a stronger and more stable polymer network, and consequently a lower swelling degree [55,56,57].

Since the degree of swelling is inversely proportional to the crosslink density, and since the concentration of the crosslinking agent remains unchanged, we believe that the difference in swelling of the gels is directly related to the pH of the synthesis [58,59]. These results are consistent with swelling data found for crosslinked galactomannan films reported by Siqueira and collaborators [54].

The results of the swelling degree show that the crosslinked Gal samples have the potential to be used in the biomedical field, especially for applications such as dressings. They can absorb fluids produced by wounds and keep the tissue moist, thus promoting the healing process [55,56].

### 3.5. Impedance Spectroscopy

**Figure 5:**
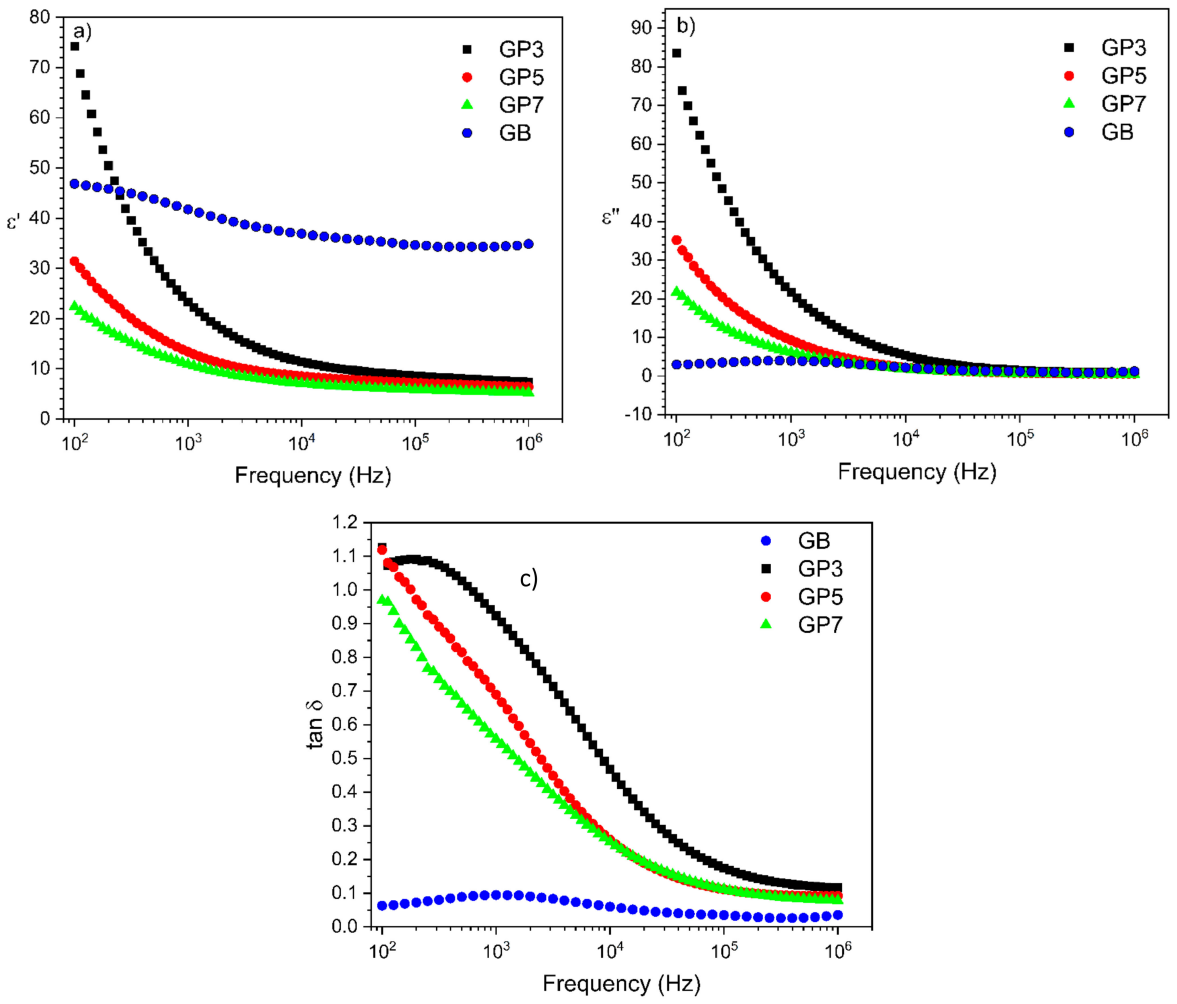
(**a**) Real and (**b**) imaginary part of complex permittivity of GB, GP3, GP5, and GP7 samples. (**c**) Loss tangent (tan δ) of GB, GP3, GP5, and GP7 samples.

Figure 5a,b present the real (*ε*’) and imaginary (*ε*″) parts of the complex permittivity for the samples at room temperature over the frequency range of 10^2^–10^6^ Hz. Comparing the dielectric response of GB and crosslinked samples (GP3, GP5, GP7), it can be observed that the former exhibits a higher *ε*’ for a wide frequency range.

Crosslinking reduces the mobility of the polymer chains due to the formation of covalent bonds and the consequent reduction in the free volume between polymer chains. This restricted mobility affects the dielectric properties of the material. It decreases the ability of the material to polarize and align in response to the electric field, which justifies why crosslinked samples present a lower *ε*’ than the GB sample [60].

**Figure 6:**
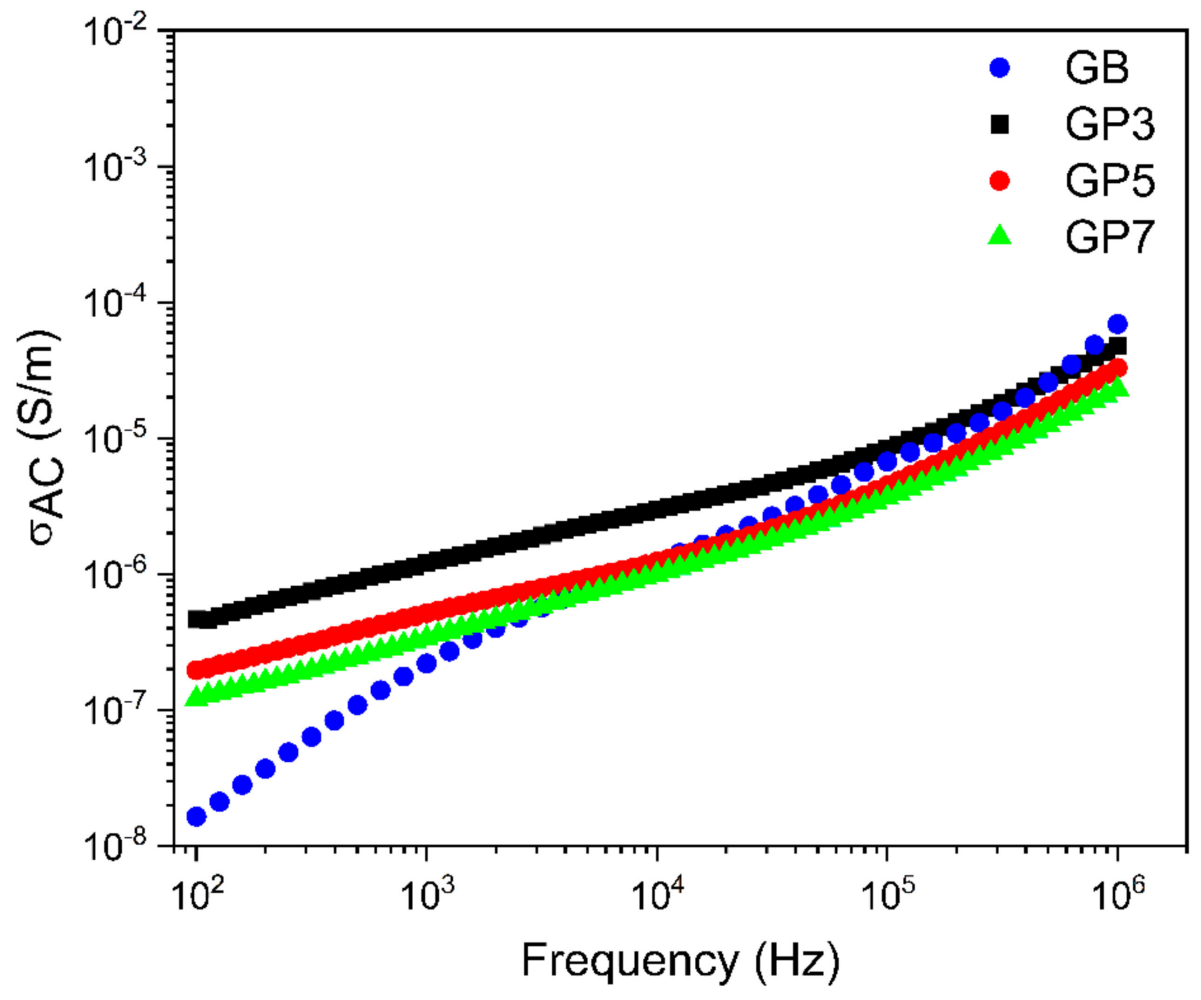
AC conductivity of GB, GP3, GP5, and GP7 samples.

Among the crosslinked samples, it is observed that sample GP7, prepared under the higher-pH medium, presents the lower *ε*’ value, for all the frequency range, which points to a higher crosslinking level. This observation is in line with the measurements of AC conductivity, presented at Figure 6. For all the frequency rang,e GP7 presents lower conductivity than GP3 and GP5. The formation of a three-dimensional network limits the movement of charge carriers, such as ions or electrons, through the material, reducing its conductivity.

Crosslinked samples present, for all the studied frequency range, a much higher dielectric loss tangent (tan *δ*) than the GB sample, as shown in Figure 5c. This behavior can be explained with the formation of covalent bonds that restricts the movement of polymer chains, resulting in increased friction and energy dissipation during the alignment and reorientation processes under an electric field.

The dielectric response was analyzed using the complex modulus formalism ($M^{*}$), as it allows a clearer separation between electrode polarization response and the other intrinsic polarization mechanisms [61]. Complex modulus ($M^{*}$) and complex permittivity ($\varepsilon^{*}$) are related through the expression $M^{*}=1/\varepsilon^{*}$, so that the real (M’) and imaginary (M’’) parts of the modulus function can be calculated from Equations (2) and (3):

(2) $$M^{\prime }=\frac{\varepsilon^{\prime }}{\varepsilon^{\prime 2}+\varepsilon^{\prime \prime 2}}$$

(3) $$M^{\prime \prime }=\frac{\varepsilon^{\prime \prime }}{\varepsilon^{\prime 2}+\varepsilon^{\prime \prime 2}}$$

**Figure 7:**
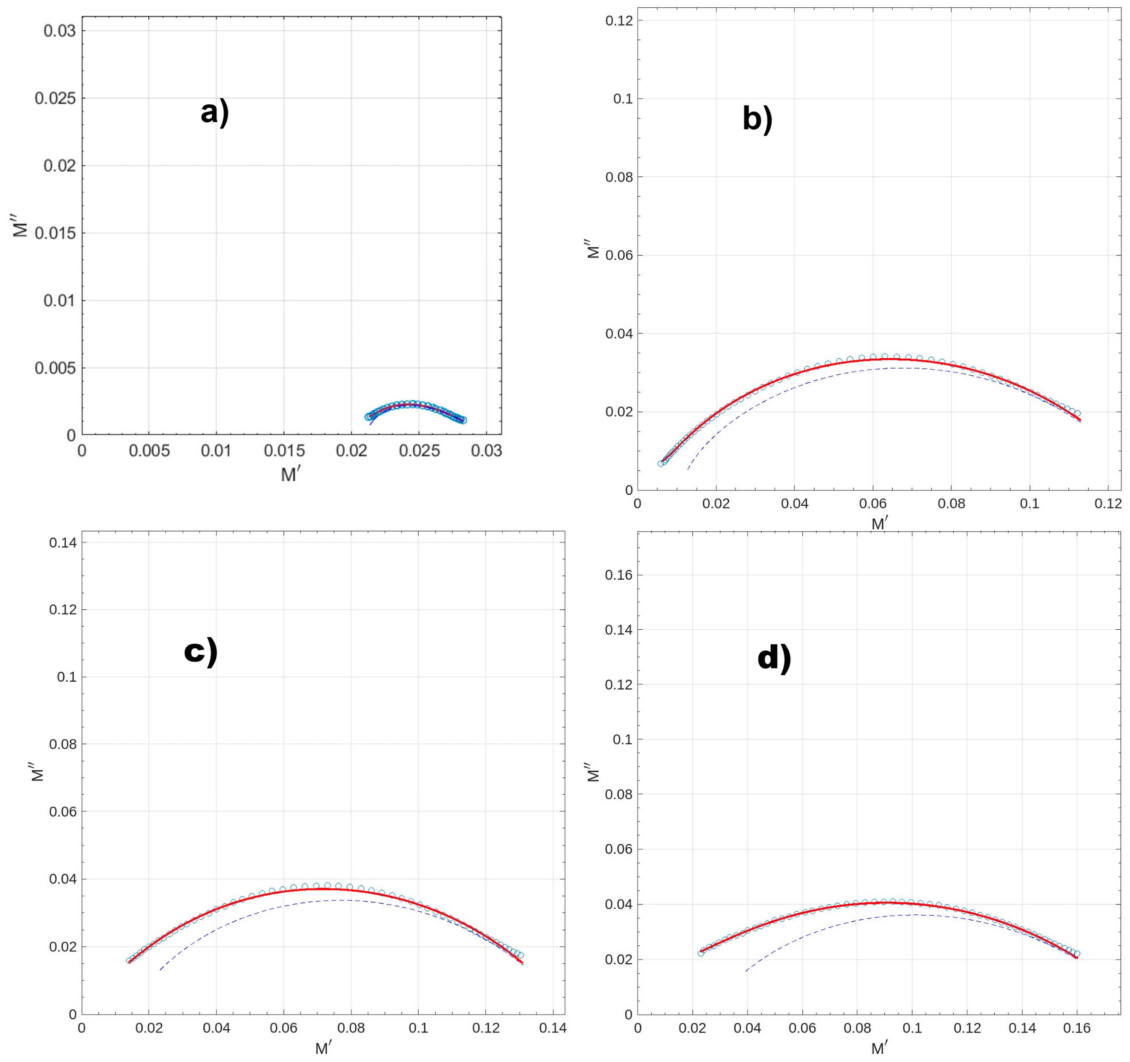
The complex modulus plane plot of (**a**) GB, (**b**) GP3, (**c**) GP5, and (**d**) GP7. Blue circles represent the experimental data, the continuous red line represents the fitting with the HN function, and the dashed line represents the fitting excluding the dc conductivity ($\sigma_{0})$.

The complex modulus plane plot, M’’ versus M’, is shown in Figure 7a–d.

The observed arcs are not centered in the M’ axis and the relaxation peaks are asymmetric, confirming that a single relaxation time described by the equation of Debye cannot be used to explain this dielectric relaxation. Therefore, the data were fitted with the Havriliak–Negami (HN) function described in Equation (4) [62]:

(4) $$M_{HN}^{*}\left(\omega \right)=\frac{1}{\varepsilon_{HN}^{*}\left(\omega \right)}=\frac{1}{\varepsilon_{\infty }+\frac{\varepsilon_{s}-\varepsilon_{\infty }}{{\left(1+(i\omega \tau_{HN}{)}^{\alpha }\right)}^{\beta }}}$$

where $\varepsilon_{\infty }$ is the dielectric constant at the high-frequency limit, $\varepsilon_{s}$ is the static dielectric constant (low-frequency limit), $\tau_{HN}$ is the relaxation time (related to the characteristic frequency $\omega_{HN}$ through the relation $\tau_{HN}\cdot \omega_{HN}=1$).

The α and β parameters define the broadness and symmetry of the arcs, with values in the range of 0<α,β≤1. For β=1, the HN equation reduces to the Cole–Cole equation, representing a broadened but symmetric Cole–Cole plot, while for α=1, the HN function, representing an asymmetric peak relaxation, reduces to the Cole–Davidson equation.

As we will describe, a non-Debye relaxation process should be considered to better explain the dielectric response of the samples. The characteristic frequency $\omega_{HN}$ is related to the loss peak frequency $\omega_{max}$ trough the relation $\omega_{max}=A\cdot \omega_{HN}$, where A is described by Equation (5). The parameters of the fitted Havriliak–Negami models for each sample are given in Table 3:

(5) $$A={\left(\frac{sin\left(sin\left(\frac{\alpha \pi }{2\pi \beta +2}\right)\right)\;}{sin\left(sin\left(\frac{\alpha \beta \pi }{2\beta +2}\right)\right)\;}\right)}^{\frac{1}{\alpha }}$$

From the fitting of the dielectric relaxation process using the HN function, we conclude that $\varepsilon_{\infty }$ is higher for GB, the non-reticulated sample, reaching a minimum value of 5.39 for the GP7 sample. The decreasing of the dielectric strength (∆ε) from 82,89 (GP3) to 33.04 (GP7) can be explained by a higher crosslinking at the higher-pH preparation medium.

Meanwhile, comparing the frequency response for reticulated and non-reticulated Gal2%, it is observed that GP samples present higher relaxation frequencies (>2 kHz) than the value observed for GB (~1 kHz). As the polymer chains become more rigid and constrained, due to crosslinking, the relaxation processes associated with the movement of dipoles or charge carriers occur at higher frequencies.

## 4. Conclusions

The pH of the media significantly influences the crosslinking reactions and the interactions between the Gal molecules. The swelling degree results confirm the chemical crosslinking of Gal. The crosslinked Gal in all pH media presents significant swelling potential and can maintain its physical structure after swelling, which makes it a stable hydrogel for water absorption. With the lowest residue content among the crosslinked samples, the crosslinked Gal at pH 3 (GP3) presented the lowest concentration of inorganic material, which, combined with its good swelling ability, results in GP3 having the highest potential for application as a hydrocolloid wound dressing.

Both crude and crosslinked Gal samples presented dielectric behavior characterized by the Havriliak–Negami model, with the crosslinked samples presenting higher dielectric losses compared to Gal due to the more rigid and constrained polymer chains generated by the crosslinking process, resulting in more energy dissipation during electric polarization. The increase in pH value resulted in higher crosslinking in the GP samples, as the decrease in the dielectric strength caused by the constrained polymer chains was intensified with the pH increase.

## Figures and Tables

**Table 1:** Polysaccharide composition and viscosity properties of *Adenanthera pavonina* L. crude Gal.

Polysaccharide	
Composition (% mol)	Mannose (52.8/pm 0.4%); Galactose (39.2/pm 1.1%); Arabinose (4.2/pm 1.0%); Xylose (1.2/pm 0.2%); Rhamnose (1.0/pm 0.3%); Fucose (0.9/pm 0.2%); Glucose (0.8/pm 0.1%).
Average intrinsic viscosity (dL/g)	9.11
*k_H_*	1.10
*M_ν_* (Da)	1.81 × 10^6^

Source: [31].

**Table 2:** Weight loss percentage and waste percentage of samples.

Sample	Weight Loss (%)				Waste (%)
	ΔW1	ΔW2	ΔW3	ΔW4	
GB	11.45	71.95	-	-	16.6
GP3	32.25	14.64	29.11	6.3	17.7
GP5	34.14	18.86	20.85	6.69	19.46
GP7	32.23	10.92	23.13	11.16	22.56

**Table 3:** Fitting of the dielectric data using Havriliak-Negami function.

Parameter	GB	GP3	GP5	GP7
$\varepsilon_{s}$	47.49	90.47	70.50	38.43
$\varepsilon_{\infty }$	33.43	7.58	6.87	5.39
∆ε =$\;\varepsilon_{s}-\varepsilon_{\infty }$	14.06	82,89	63.63	33.04
$\tau_{HN}$(s/rad)	4.16 × 10^−4^	1.47 × 10^−3^	3.97 × 10^−3^	2.89 × 10^−3^
$f_{max}$(Hz)	973.7	8.29 × 10^3^	2.33 × 10^3^	2.72 × 10^3^
α	0.864	0.919	0.766	0.688
β	0.435	0.621	0.748	0.732
$\sigma_{0}$(S/m)	7.97 × 10^−9^	2.86 × 10^−7^	9.65 × 10^−8^	7.34 × 10^−8^

## Data Availability

The original contributions presented in this study are included in the article. Further inquiries can be directed to the corresponding author(s).
